# Near-Infrared Spectroscopy with Supervised Machine Learning as a Screening Tool for Neutropenia

**DOI:** 10.3390/jpm14010009

**Published:** 2023-12-21

**Authors:** José Joaquim Raposo-Neto, Eduardo Kowalski-Neto, Wilson Barros Luiz, Estherlita Almeida Fonseca, Anna Karla Costa Logrado Cedro, Maneesh N. Singh, Francis L. Martin, Paula Frizera Vassallo, Luciene Cristina Gastalho Campos, Valerio Garrone Barauna

**Affiliations:** 1 Department of Health Sciences, State University of Santa Cruz, Ilhéus 45662-900, Brazil; ekneto@uesc.br; 2 Laboratory of Applied Pathology and Genetics, State University of Santa Cruz, Ilhéus 45662-900, Brazil; wbluiz@uesc.br (W.B.L.); estherlita.bio@uesc.br (E.A.F.); akclcedro.bio@uesc.br (A.K.C.L.C.); 3 Department of Biological Science, State University of Santa Cruz, Ilhéus 45662-900, Brazil; 4 Biocel UK Ltd., Hull HU10 6TS, UK; mnsingh@biocel.uk (M.N.S.); flm13@biocel.uk (F.L.M.); 5 Chesterfield Royal Hospital, Chesterfield S44 5BL, UK; 6 Department of Cellular Pathology, Blackpool Teaching Hospitals NHS Foundation Trust, Whinney Heys Road, Blackpool FY3 8NR, UK; 7 Clinical Hospital Universidade Federal de Minas Gerais, Belo Horizonte 31270-901, Brazil; paulafv@ufmg.br; 8 Department of Physiological Science, Federal University of Espírito Santo, Vitória 29932-540, Brazil; valerio.barauna@ufes.br

**Keywords:** neutropenia, neutrophils, NIR spectroscopy, machine learning, fingertip

## Abstract

The use of non-invasive tools in conjunction with artificial intelligence (AI) to detect diseases has the potential to revolutionize healthcare. Near-infrared spectroscopy (NIR) is a technology that can be used to analyze biological samples in a non-invasive manner. This study evaluated the use of NIR spectroscopy in the fingertip to detect neutropenia in solid-tumor oncologic patients. A total of 75 patients were enrolled in the study. Fingertip NIR spectra and complete blood counts were collected from each patient. The NIR spectra were pre-processed using Savitzky–Golay smoothing and outlier detection. The pre-processed data were split into training/validation and test sets using the Kennard–Stone method. A toolbox of supervised machine learning classification algorithms was applied to the training/validation set using a stratified 5-fold cross-validation regimen. The algorithms included linear discriminant analysis (LDA), logistic regression (LR), random forest (RF), multilayer perceptron (MLP), and support vector machines (SVMs). The SVM model performed best in the validation step, with 85% sensitivity, 89% negative predictive value (NPV), and 64% accuracy. The SVM model showed 67% sensitivity, 82% NPV, and 57% accuracy on the test set. These results suggest that NIR spectroscopy in the fingertip, combined with machine learning methods, can be used to detect neutropenia in solid-tumor oncology patients in a non-invasive and timely manner. This approach could help reduce exposure to invasive tests and prevent neutropenic patients from inadvertently undergoing chemotherapy.

## 1. Introduction

Neutropenic patients are not recommended for chemotherapy since it induces further neutrophil count decreases [1]. Chemotherapy treatments for cancer are well-known to be associated with a depletion of white blood cells, particularly neutrophils, causing a condition known as chemotherapy-induced neutropenia. Lower neutrophil count levels have been linked to lower survival rates, lower quality of life, and a higher risk of opportunistic infections [2].

In clinical practice, the cut-off value of a neutrophil count (1500 cells/mm^3^) is typically used to exclude, delay, or lower the dosages of a chemotherapy session [3]. In this scenario, neutropenic patients are further classified as having lower counts (higher overall risk) or higher neutrophil counts (lower overall risk). In oncologic patients, a neutrophil count of fewer than 500 cells/mm^3^ can indicate a poor prognosis, a shorter life expectancy, and a high risk of death [4].

Despite being a cornerstone in clinical practice, a complete blood count (CBC) assessment might impose some costs and challenges [5]. For example, an Italian study conducted in Bari, with approximately one million inhabitants and an estimated price for a blood count test of USD 3.14, resulted in a total cost for undertaking this test of USD 560,000. Considering the entire national territory of Italy, the estimated cost surpasses USD 20 million per year for outpatients at public hospitals. However, the laboratory costs for other cases, such as patients in hospitals and private clinics, are substantially greater than this estimate [6]. Beyond the costs, a peripheral venous puncture can significantly increase the risk of hemorrhage in patients with low platelet levels or coagulopathies [7]. Furthermore, a peripheral blood count cannot be collected quickly due to patient dehydration, anatomic abnormalities of peripheral veins, or the inability to maintain intravascular patency after a puncture [8].

To overcome these challenges and provide a safe chemotherapy procedure, a quick, non-invasive, and on-site point-of-care approach to detect low neutrophil counts in solid-tumor oncologic patients could aid in patient selection for chemotherapy. The critical element in reducing neutropenia-related mortality is early diagnosis before the onset of infection and fever, resulting in a reduced need for hospitalization and a faster neutrophil recovery. One major obstacle faced in managing chemotherapy-induced neutropenia (CIN) is the timely diagnosis of neutropenia before the onset of infection. Timely diagnosis would provide a critical window of opportunity for appropriate clinical intervention (with G-CSF and antibiotics). The number one challenge in diagnosing CIN is the patient’s ability to identify the signs and symptoms of CIN and realize that they are seriously ill and need to report immediately to the appropriate cancer care or hospital ward [9]. It is essential to educate all cancer patients and chemotherapy patients about the risks of CIN before beginning treatment. Thus, a method to detect low neutrophil counts in oncologic patients might help better select patients for chemotherapy.

The technique of near-infrared (NIR) spectroscopy was first described by William Herschel in 1800. The spectroscopy principles consist of using a specific wavelength light device emitter to characterize the molecular composition of the sample qualitatively and quantitatively [10]. As the light photons interact with the target sample, a fraction of the wave energy is absorbed, dispersed, and reflected [11]. In this context, NIR spectroscopy consists of a technique that uses a shorter wavelength interval with a higher frequency light device emitter. The first documented NIR application was to continuously monitor cerebral tissue oxygen saturation made by Jobsis in 1977 [12]. A recent study by Chaves et al. applied NIR spectroscopy to the patient’s thenar eminence to investigate the impacts of continuous venovenous hemodiafiltration on the microcirculation in patients with acute kidney injury [13].

The achievement of an early diagnosis is essential for success in treating any disease, including in the oncological field. In the absence of clinically detectable signs and symptoms or efficient screening programs for several diseases, new parameters need to be evaluated. New ideas and techniques are evolving to fill this gap, which would be impossible with traditional methods. In the last two decades, there has been scientific interest in vibrational spectroscopy (VS), an analytical method potentially applicable to fingertip tissue. While spectroscopy investigates the interaction between electromagnetic radiation (light) and matter, VS identifies the molecular structure of the analyzed sample through its vibrational characteristics interacting with a beam source. The revealed spectrum represents a faithful representation of the unique molecular characteristics investigated. This rapid, non-destructive, minimally invasive, and relatively inexpensive technique has shown to be promising in detecting biomarkers in different samples [14]. Hence, with the emerging progress in technology and machine learning, the analysis and classification of multiple parameters derived from spectroscopic analysis have been facilitated, and its potential exponentially elevated.

Therefore, this study aims to assess whether NIR spectroscopy in the fingertip coupled with supervised machine learning classification algorithms can detect neutropenic patients and develop a quick and practical measurement tool contributing to clinical decision making in the oncology field.

## 2. Materials and Methods

### 2.1. Participants and Samples

Blood count samples from 75 patients were obtained by venipuncture using sodium citrate vacuum tubes in hospital Santa Casa de Misericordia de Itabuna, BA, Brazil. The inclusion criteria to participate in the study were >18 years old, diagnosed solid-tumor oncology patients, and the complete blood count performed on the same day. It is common for cancer patients to have their blood cells counted periodically to decide whether or not they can undergo the next chemotherapy session. The exclusion criteria were <18 years old and hematological tumor patients. All patient clinical data were obtained from the medical records. Patients were assigned to the neutropenic group (NG) if CBC < 1500 neutrophil cells/mm^3^ or were assigned to the normal neutrophil group (NNG) if CBC ≥ 1500 neutrophil cells/mm^3^. The patient demographic data are reported in Table 1 and the Section 3.

This study was carried out in agreement with the Helsinki Declaration. The Local Ethics Committee granted full ethical approval for the investigation at the State University of Santa Cruz (CAEE: 48245921.0.0000.5526). All the volunteers signed the informed consent form before being included in the study.

### 2.2. Differential Stain of Blood Cells

Blood smears from four random neutropenic patients and four normal individuals were stained with the Instant-Prov kit (Newprov, Pinhais, Brazil) according to the manufacturer’s instructions. The slides were examined under common light microscopy at 100× magnification.

### 2.3. NIR Spectroscopy

The NIR spectra were taken non-invasively (without blood sample collection). For spectral acquisition, the patient’s index finger from the right hand was cleaned with a 70% ethanol *v*/*v* solution and positioned over a portable NIR spectroscopy (MicroNir ES 1700, VIAVI, Chandler, AZ, USA) to obtain the patient’s spectra, as previously published [15]. The spectral range obtained was from 908 to 1676 nm in absorbance mode with a 6.2 nm resolution and 125 different wavelengths registered. Sample spectra were recorded in triplicate (n = 225 spectra).

#### 2.3.1. NIR Chemometric Analysis

This study’s entire chemometric analysis pipeline was developed in a Python v3.10.12 environment. The raw spectra were pre-processed with a 7-point Savitzky–Golay smoothing method with a second-order polynomial. The Savitzky–Golay smoothing method is a pre-processing method to smooth noisy spectral data while preserving important features. It is widely used to avoid the common pitfall of moving averages and linear filters to shrink relevant details from data. It is commonly applied to spectroscopy and other types of signal processing. Savitzky–Golay operates on small, overlapping windows of the data, and a polynomial function is fitted to the data within each window size. In each window, a polynomial of a specified degree is fit to the data points. The degree of the polynomial can be an optimized parameter based on the characteristics of the data. The coefficients of the polynomial function are determined by minimizing the sum of the squared differences between the actual data points and the values predicted by the polynomial. Next, the coefficients obtained from the polynomial fitting are convolved with the original data. This convolution is essentially a weighted moving average in which the coefficients of the fitted polynomial determine the weights. The convolution operation smoothens the data by averaging noise and preserving important features, such as peaks and valleys. Other pre-processing sequences were also tried, although the presented one showed the best classification performance. The Hotelling T^2^ × Q-residuals were applied for outlier detection (Supplemental Figure S1), and 1 patient from the NNG was removed from the dataset due to low-quality collection issues (Supplemental Figure S2). The choice rationale of machine learning models utilized in this study aimed to include linear and non-linear models with different complexity profiles.

To correct for the class imbalance between NG and NNG groups, a penalization factor hyperparameter was included in each of the 5 tested algorithms. The value for the penalization factor was defined as 3. The number represents the inverse of the absolute prevalence of neutropenic patients in the study cohort. A penalization factor of 3 was selected based on the approximate integers greater than and lower than the inverse proportion of samples in the minority class (NG) and majority class (NNG). The penalization factor was chosen based on a grid search hyperparameter optimization method with values of penalization factors ranging from 1 to 5. The value of 3 gave the lowest bias–variance trade-off and optimal statistical performance between training and validation metrics in our dataset.

#### 2.3.2. Linear Discriminant Analysis

Linear discriminant analysis (LDA) is a supervised pattern recognition tool that is a set of data with already known classes used to train the model, and later this model is used to classify new data based on the selected characteristics [16]. This method uses the principle of maximizing variation between classes and minimizing variation within the same class to create a linear decision boundary between classes. It is important to highlight that LDA assumes that the independent variables have a normal distribution [16]. Through LDA, new samples can be classified into one of the pre-established categories based on the observed characteristics. This tool is widely used in areas such as pattern recognition, biometrics, and data analysis. Linear discriminant analysis (LDA) is a supervised machine learning algorithm for dimensionality reduction and classification. LDA is designed to maximize the separation between different data classes. This algorithm assumes that each class is normally distributed and has the same covariance matrix. When these assumptions are met, LDA can perform optimally compared to other non-parametric machine learning algorithms. Because LDA makes assumptions about data distribution, it can be suitable for scenarios where the number of observations is limited. An important limitation is that LDA is sensitive to outliers because it relies on means and covariance matrices. Outliers can significantly affect these statistics and, consequently, the performance of LDA. Another pitfall of LDA is the linear decision boundary between classes. In cases where the true boundary is highly non-linear, other methods, like support vector machines (SVM) or non-linear algorithms, might be more appropriate. Further, while LDA maximizes inter-class distance, it does not explicitly consider variability within each class. In situations where intra-class variability is high, other techniques, like quadratic discriminant analysis (QDA), might perform better.

#### 2.3.3. Logistic Regression

Due to the interpretability of the logistic regression (LR) algorithm, its low computational cost, and its weak tendency to over-fit training data, it is a suitable classification algorithm to deal with data that suffers from high dimensionality matrix space, where the number of independent variables is higher than the number of samples in the dataset. LRs consist of a general closed-form parametric equation of a linear regression model with a sigmoid function transformation applied to the prediction values, transforming probability values between zero and one. The coefficients for each independent variable in logistic regression can be determined by the single value decomposition (SVD) matrix method. Each coefficient represents the impact of the independent variable on the final model prediction. SVD is a powerful matrix factorization technique widely used in machine learning. It can offer several advantages: (1) effectively determining coefficients in multi-collinearity problems; (2) provide a numerically stable approach to solving linear systems; and (3) enable dimensionality reduction in machine learning algorithms, including LR and regularization methods. Its versatility and low computational cost make it a valuable tool for understanding and analyzing complex relationships within data.

#### 2.3.4. Support Vector Machines

Support vector machines (SVMs) were initially proposed by Cortes and Vapnik in 1995 [17]. To tackle limited sample sizes and non-linear and high-dimensional pattern recognition problems, SVMs have numerous advantages. SVM models depict examples as spatial locations with the greatest possible distinct spacing between instances of different classes. The same space maps new instances, which are then projected to belong to a class based on which side of the interval they fall. Input vectors for SVMs are non-linearly transferred to a very large feature space. SVMs identify the ideal hyperplane that has the most significant potential distance between the two classes. The data points closest to the separating hyperplane among the sample points in the training dataset are referred to as “support vectors” for linear separability. Finding a hyperplane that can completely segregate both sorts of data for practical activities is challenging. Allowing the SVM to be incorrect for a few samples by adjusting a penalty parameter factor to the objective optimization function is one way to solve this issue. This solution can be applied mainly with class imbalance tasks. In SVMs, the penalty parameter, often denoted as C, is a crucial hyperparameter that controls the trade-off between achieving a low training error and a low testing error. The significance of the penalty parameter in SVM can be explained in the context of the soft-margin SVM and its impact on the decision boundary. The SVM algorithm aims to find a hyperplane that separates different classes while maximizing the margin between the hyperplanes. In real-world problems, data may not be perfectly separable. The soft-margin SVM introduces the penalty parameter C to allow for some misclassification (points falling on the wrong side of the margin or even within it). When C is small, the model is more tolerant of misclassifications. It prioritizes achieving a wider margin, even if it allows some training points to be misclassified. This is useful when dealing with noisy or overlapping data. Otherwise, when C is a large value, the model becomes less tolerant of misclassifications. It emphasizes correctly classifying as many training points as possible, even if it means having a smaller margin. This can lead to a more complex decision boundary. Thus, the penalty parameter acts as a regularization term. A smaller C imposes a more robust regularization, resulting in a simpler model with a larger margin. A larger C reduces the regularization, allowing for a more complex model that fits the training data closely.

#### 2.3.5. Random Forest

Due to the complexity and non-linearity of biological spectroscopic information, tree-based models often display robust and stable behavior compared to linear feature selection models, such as LR. Random forests are a collection of tree predictors where each tree depends on the values of a random vector sampled independently and with the same distribution for all the trees in the forest. As the number of trees in the forest increases, the generalization error converges to a limit. The strength of the individual trees in the forest and the correlation between them determine the accuracy of a forest of tree classifiers. Each node is split using a random selection of features, producing error rates more resilient to noise. Internal estimates keep track of loss function, strength, and correlation; they demonstrate how the splitting process responds to an increase in the number of features [18]. In the context of machine learning, especially ensemble methods and random forests, this concept refers to the robustness of the model in handling noisy or irrelevant features in the dataset. Random forests naturally can mitigate the impact of noise and outliers, leading to more accurate and stable predictions. The ensemble nature of random forests allows them to average out the predictions of individual trees. Noisy predictions from individual trees are mitigated when aggregated across the ensemble, leading to a more robust and stable prediction.

#### 2.3.6. Multilayer Perceptron

In the 1950s, Rosenblatt generated a framework for an artificial neural network (ANN) called a “Perceptron” that was modeled after biological brain networks [19]. An output is generated by an activation function in ANNs by combining the input signal (x), weights (w), and bias term (b). Training the weight parameters allows the perceptron to optimize the model. A basic perceptron, however, can only resolve linear issues. Hidden layers are necessary for a network used to solve non-linear problems. Back-propagation neural network (BNN) technology was invented by Rumelhart et al. in 1986. The fundamental tenet of a BNN is that learning includes both forward signal and backward error propagation. A typical ANN structure has a large number of artificial neurons stacked on top of one another. Multilayer perceptron (MLP) is a specific architecture of neural networks encompassing input layers, a set of hidden layers, and output layers that optimize based on backward propagation and gradient descent. Multilayer perceptions (MLPs), a subtype of artificial neural networks, are widely used in machine learning tasks today due to several factors contributing to their effectiveness and versatility. MLPs can model complex, non-linear relationships between input and output features. The presence of multiple layers and non-linear activation functions allows them to capture intricate patterns and representations in the data. Therefore, an MLP algorithm with sufficient neurons in the hidden layers can effectively approximate continuous function, adding to its versatility.

### 2.4. Statistical Analysis

Neutrophil count (normal neutrophil count group versus neutropenic group) was analyzed using a Kolmogorov–Smirnov test to check for normality distribution. Statistical significance was considered if *p* < 0.05. In the next step, the Mann–Whitney U test was applied to check if neutrophil counts between NG and NNG groups were statistically significantly different. To carry out the statistical data analysis, Python v3.9 was used with the scipy v1.11.0 library.

## 3. Results

**Figure 1:**
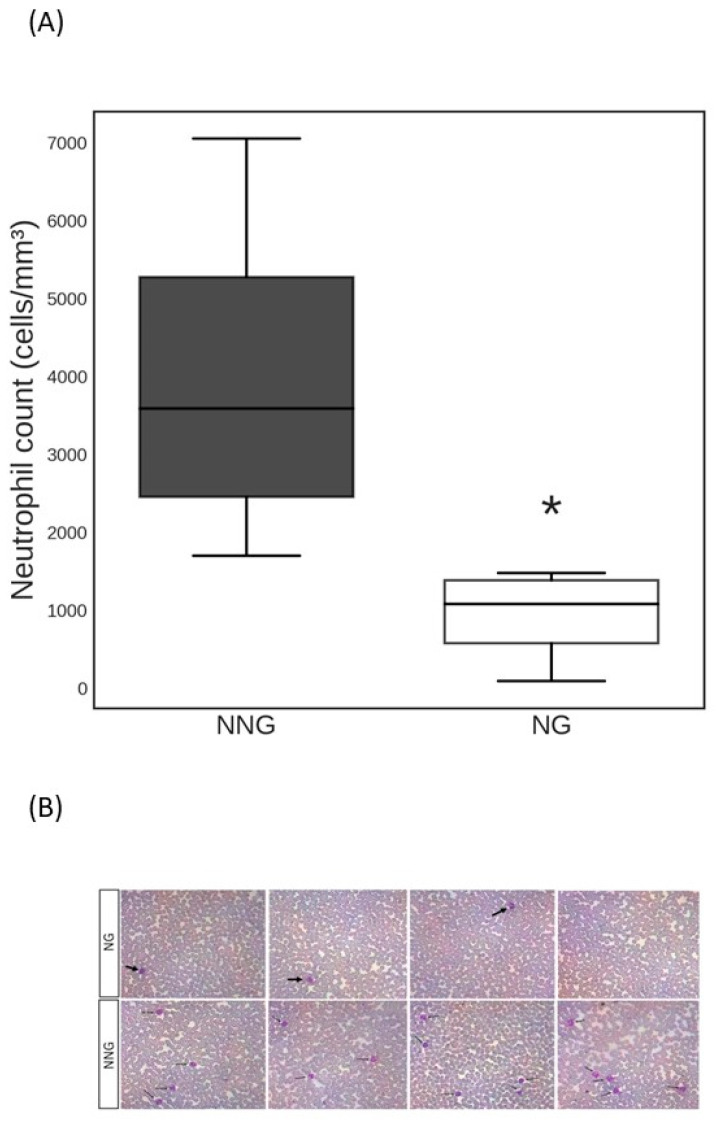
(**A**) Boxplot of neutrophil count values per group. Interquartile ranges from the 25th to 75th comprise the box body. The black horizontal line presents the median in the center of the box. The boxes’ whiskers show the distribution. (**B**) Representative image of normal and neutropenic blood smear. Black arrows indicate the neutrophil. * *p* < 0.05 vs. NNG.

The 75 solid-tumor oncologic patients were separated into two groups: the normal neutrophil count (NNG) group (≥1500 cells/mm^3^; n = 51) and the neutropenic (NG) group (<1500 cells/mm^3^; n = 24). The boxplot of neutrophil count per group is displayed in Figure 1A, while Figure 1B is a representative image of neutropenia by blood smear. In our cohort, five patients (20%) were classified as severe neutropenic (neutrophil count < 500 cells/mm^3^), four patients (16%) as moderate neutropenic status (neutrophil count between 500 and 999 cells/mm^3^), and fifteen patients (62.5%) as mild neutropenic patients (neutrophil counts between 1000 cells/mm^3^ and 1499 cells/mm^3^). There was no difference in age for the NG (53 ± 13 years) and NNG groups (53 ± 12 years; *p* > 0.05). The cohort comprised 55 (73%) female and 20 (27%) male patients. There was no statistically significant difference in sex distribution between groups (females: NG, 75% vs. NNG, 72%). The female preponderance can be explained by the higher prevalence of breast cancer patients in our sample (54%). The other two most prevalent neoplastic disease types in our cohort were prostate cancer (8%) and uterine cancer (8%). Table 1 summarizes patients’ descriptions for NG and NNG.

**Figure 2:**
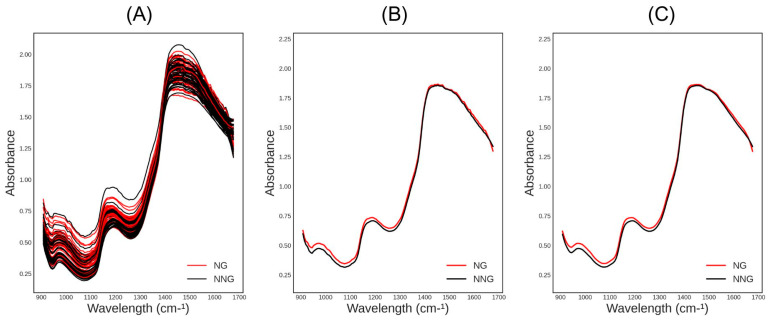
(**A**) NIR raw spectra of the 75 samples; (**B**) average NIR raw spectra per group; (**C**) average NIR pre-processed spectra per group.

The averaged raw NIR spectra of each group are depicted in Figure 2A. The red line represents the NG and the black line represents the NNG. Figure 2B shows the average spectra per group after pre-processing.

To develop a high-sensitivity classifier, five supervised ML algorithms were trained: LDA, LR, SVMs, RF, and MLP. The pre-processed spectral data were divided into groups using the Kennard–Stone uniform sample selection technique, where 70% of the samples were used for training/validation and 30% for the test set.

**Figure 3:**
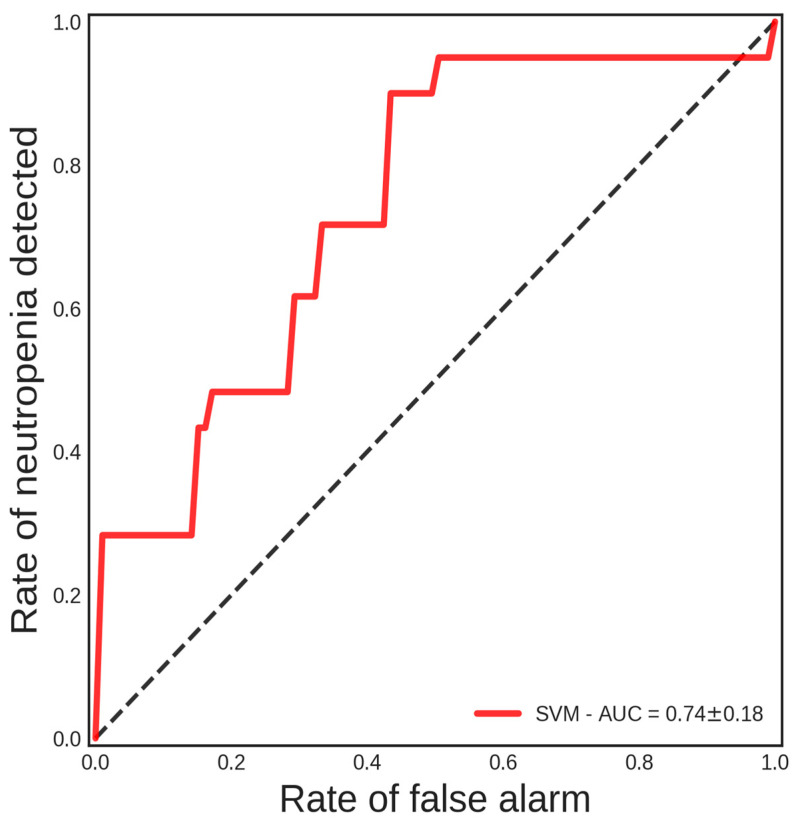
Five-fold stratified cross-validation ROC curve of the SVM algorithm. The red-colored curve represents the mean ROC curve from the five folds.

To select the best algorithm for the test set, a stratified 5-fold cross-validation regimen was utilized. The effectiveness of the models’ validation metrics was assessed by measuring sensitivity, negative predictive value, accuracy, and AUC. Sensitivity is the percentage of correctly classified positives; negative predictive value is the percentage of correctly classified negatives between all negative model predictions; accuracy is the total number of samples correctly identified while taking true and false negatives into account; and AUC is the area under the ROC curve. Table 2 shows the highest sensitivity and negative predictive value metrics in bold that correspond to the SVM algorithm. Thus, the SVM produced the best validation results. The cross-validation ROC curve for SVM is evidenced in Figure 3.

**Figure 4:**
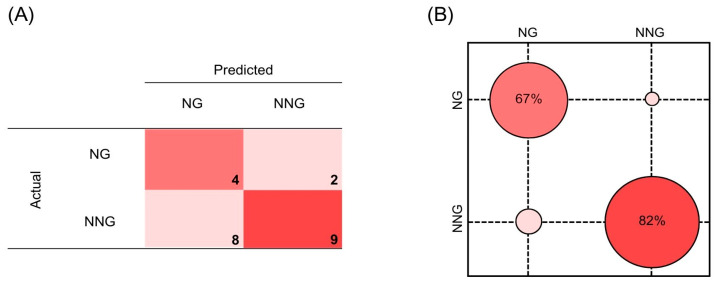
(**A**) Confusion matrix of SVM predictions on the test set. (**B**) Confusion ball representation of sensitivity (67%) and negative predictive value (82%). All data refer to the test set predictions.

Subsequent predictions of the SVM model on the test set displayed 67% sensitivity, 82% NPV, and 57% accuracy. The confusion matrix for the SVM test set predictions is shown in Figure 4.

## 4. Discussion

**Figure 5:**
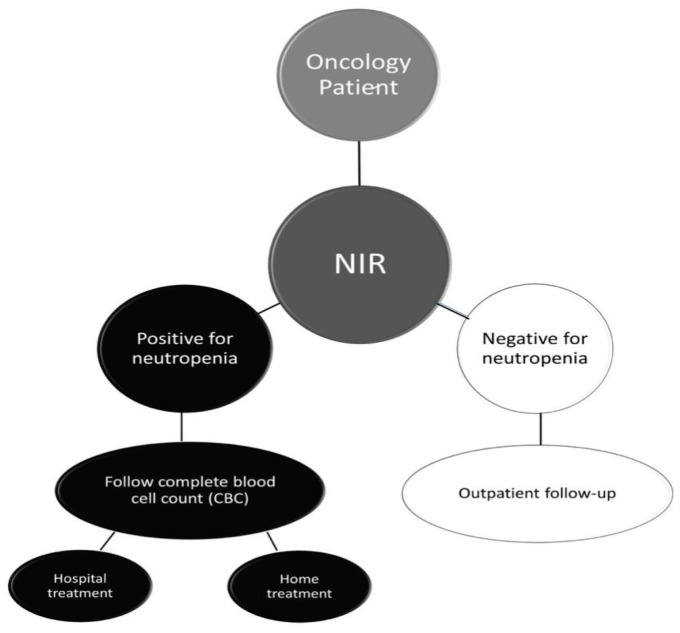
Scheme of NIR application in medical routine. The approximate procedure time is <5 min, without using blood collection material (syringe, needle) or performing venipuncture.

Our results demonstrate that fingertip NIR spectroscopy coupled with machine learning effectively classifies patients according to the established cut-off point for neutrophil count levels (Figure 5). Furthermore, this classification model was obtained using a single machine learning algorithm (SVM) without further complex and black box workflow steps. Therefore, NIR spectroscopy is a quick and safe tool for assessing neutropenia in a solid-tumor oncologic patient setting. This technology displays its potential, especially in remote and vulnerable areas, where access to healthcare professionals and widely available laboratory resources are scarce, contributing to the efficiency of the health system and improving resource allocation [20].

Neutrophils are an important type of immune cell in several contexts of diseases related to the activation or orchestration of an effective immune response [21]. Although elevated neutrophil counts can indicate inflammation or infection burden, they can also point to a proliferative blood and bone marrow disorder. Otherwise, low absolute neutrophil counts can generally indicate viral infection, autoimmune disease, or a neoplastic process [22]. The neutrophil count gains significant importance in the latter group. One of the most critical conditions in an oncologic patient is febrile neutropenia, a clinical condition characterized by the acute onset of fever and a neutrophil count below the lower levels of normality. The morbi-mortality of febrile neutropenia can range from numbers as high as 20%, and the total hospital costs with cancer attributed to febrile neutropenia can even reach 40% [2,23]. Therefore, oncological decision making regarding initiating or continuing chemotherapy must be strictly controlled. Virtually all patients should undergo a complete blood count test before beginning antineoplastic schemes, as it is well known that most patients experience a neutrophil count decrease after chemotherapy administration [1]. Therefore, neutropenia has a negative impact on the delivery of planned chemotherapy regimens, with dose reductions and treatment delays that compromise long-term clinical outcomes and cancer treatment [3]. No monitoring system is available to detect neutropenia at the time of onset in an at-home or near-patient setting [24].

Additionally, the impact of the neutrophil measurement tool presented in this article goes even further from a cost perspective, including reduing the production of biological residues, minimizing psychological stress, and relieving the painof the patients. A complete blood count (CBC) requires admission to the hospital for access to gold standard equipment and hospital personnel to complete the blood draw and subsequent analysis. If a blood test reveals low neutrophils, the patient is admitted to the hospital for further treatment with intravenous antibiotics, growth factors, and blood transfusions [24]. Neutrophil measurement tools are widely accessible, non-invasive, on-premises, point-of-care, cost-effective, and sustainable, as NIR spectrometers are not disposable devices, making the presented results relevant for further evaluation and investigation with a larger sample size.

Due to the high negative predictive value of the solution tool proposed in this paper, a framework suggested by the authors states that oncology patients are initially screened with NIR spectroscopy. A negative NIR result displays elevated confidence to assure outpatient follow-up, as illustrated in Figure 5. Otherwise, if the NIR screening test is positive, the patient should undergo a complete blood count to determine in-hospital or out-of-hospital care. In this context, our study presents a potentially effective and robust methodology for predicting neutropenic patients faster than the gold standard method (CBC), without being invasive and with lower overall costs than traditional blood count tests, which can guide healthcare in chemotherapy decision making.

Extrapolating the good statistical metrics obtained in this study, the economic viability of the proposed neutrophil measurement tool showed that on a larger scale, NIR coupled with machine learning models could improve the arguments for adopting the technique. In addition, the classification model provided stable sensitivity and NPV values. It is also important to note that sensitivity and NPV are the most suited evaluation metrics in this study scenario, as triage tests require high sensitivity, and the consequences of administering chemotherapy agents to neutropenic patients could be catastrophic and irreversible. Another point to consider is that the ML model design can be easily deployed to a cloud-based infrastructure, serving different geographic regions in real time and on a large scale. Thus, NIR spectroscopy associated with machine learning in medical practice can safely improve decision making, reducing the public health system resource burden and improving patients’ quality of life [25,26,27].

Integrating NIR spectroscopy with machine learning as a screening tool in medical diagnostics can have significant implications for healthcare [26,27]. The proposed NIR + ML screening tool can enable early detection of neutropenia by analyzing subtle changes in biological fingertip tissue that may precede noticeable symptoms. This can improve clinical assistance and reduce the delay in therapeutic support implementation. Thus, early detection often leads to more effective treatment, better prognosis, and the potential for reduced healthcare costs. Another implication is that NIR is non-invasive, allowing for data collection without invasive procedures or sample collection. Non-invasive screening tools are more patient-friendly, potentially increasing patient compliance and participation in routine screenings. However, portable NIR devices combined with ML algorithms could facilitate point-of-care testing, bringing diagnostics closer to the patient. Rapid and on-site diagnostics can lead to quicker decision making by healthcare professionals, particularly in resource-limited settings. In the broader healthcare context, ML algorithms can analyze NIR data to identify individualized biomarkers and patterns, contributing to the development of personalized treatment plans. This can help tailor medical interventions based on individual patient characteristics, enhancing treatment efficacy and reducing the risk of adverse effects. The conclusions of this study could help in future studies about continuous monitoring using NIR + ML to provide real-time data on disease progression and treatment response. This could allow timely adjustments to treatment plans, leading to more effective management of chronic conditions. Furthermore, the results from our study could enhance research opportunities in machine learning applied to healthcare. NIR + ML as a screening tool can aid in large-scale data collection, foster research and the discovery of new biomarkers, and lead to a better understanding of disease mechanisms supporting the development of novel therapies and interventions.

The main limitation of this study is the small size of the cohort used. Another potential limitation is that the NIR + ML triage tool was only tested for a solid-tumor patients cohort, raising concern about the generalizability of the solution from hematological tumor and non-oncologic populations. NIR spectroscopy typically covers a limited spectral range, and certain important information may fall outside this range. This can be a possible source of bias if the most important features for classification are not well represented in the spectral data range. The model may not perform as optimally as expected. Another possible limitation is that the effectiveness of the classification model may be impacted by variations in the samples used for training. Thus, if the training dataset does not adequately capture the variability present in real-world samples, the model may not generalize well. Also, NIR may have inherent variability or drift over time. Variability in spectral acquisition performance can introduce bias, especially if not properly calibrated. Furthermore, pre-processing NIR spectral data can be challenging, and choosing pre-processing methods can influence model performance. Bias may arise if pre-processing steps are not standardized or if they introduce artifacts that affect the model’s ability to generalize. A limitation of NIR spectra stability is the influence of external factors, such as lighting or temperature changes, which can affect NIR measurements. Addressing these limitations and potential bias sources requires careful consideration in designing, implementing, and validating NIR-based machine learning models. Rigorous validation, diverse and representative datasets, and transparency in model development are essential steps to mitigate bias and ensure reliable and unbiased results in real-world applications [28]. Despite the limitations described, as a proof of concept, we successfully highlighted the potentiality and advantages of the NIR spectroscopy from the fingertip for neutrophil count evaluation.

## 5. Conclusions

In the present study, NIR spectroscopy associated with machine learning distinguished solid-tumor oncology patients’ fingertip samples based on neutrophil values with a sensitivity of 67% and negative predictive value of 82%. Fingertip biochemical complexity is reflected in the NIR data, and it needs an effective statistical analysis to extract relevant information. Our study makes significant contributions to the existing literature on non-invasive diagnostics. First, integrating NIR + ML represents an advancement in non-invasive diagnostic technology. This study uncovered the capabilities of this combined approach and how it compares to or complements existing non-invasive diagnostic methods. Also, this study helped by providing evidence of the accuracy and reliability of the NIR + ML screening tool. This includes showcasing the sensitivity, specificity, and overall performance metrics compared to traditional diagnostic methods. Furthermore, the literature on non-invasive diagnostics often focuses on specific medical fields. A study utilizing NIR + ML can contribute by demonstrating the versatility of this approach across various medical domains. A significant contribution is that the proposed NIR + ML screening tool can be easily integrated into existing diagnostic workflows. This includes considerations for feasibility, ease of use, and potential enhancements to current diagnostic practices. This proposed technique can contribute to clinical decision making in the oncology field.

## Figures and Tables

**Table 1:** Patients’ cohort information per group.

	NG	NNG
Total (n)	24	51
Sex % (M/F)	25/75	28/72
Age range	24–76	29–80
Average age	53 ± 13	53 ± 12

**Table 2:** Five-fold stratified cross-validation metrics for the five algorithms used in this study. NPV = negative predictive value. ROC AUC = area under the receiver operator characteristics (ROC) curve.

Model	Accuracy	Sensitivity	NPV	ROC AUC
LDA	68%	48%	73%	73%
LR	70%	35%	72%	76%
SVM	62%	85%	89%	74%
RF	66%	46%	72%	77%
MLP	66%	28%	69%	71%

## Data Availability

The datasets generated during and/or analyzed during the current study are available from the corresponding author upon request.

## Supplementary Materials

The following supporting information can be downloaded at https://www.mdpi.com/article/10.3390/jpm14010009/s1, Figure S1: Hotelling T^2^ × Q plot evidencing one outlier (patient id = 107); Figure S2: Outlier spectra versus average spectra per group (NG × NNG).

## References

[1] Crawford J., Dale D.C., Lyman G.H. (2004). Chemotherapy-Induced Neutropenia. Cancer.

[2] Lyman G.H., Michels S.L., Reynolds M.W., Barron R., Tomic K.S., Yu J. (2010). Risk of Mortality in Patients with Cancer Who Experience Febrile Neutropenia. Cancer.

[3] Gupta A. (2019). Management of Chemotherapy Induced Neutropenia—An Unmet Clinical Need. Am. J. Biomed. Sci. Res..

[4] Cao X., Ganti A.K., Stinchcombe T., Wong M.L., Ho J.C., Shen C., Liu Y., Crawford J., Pang H., Wang X. (2020). Predicting Risk of Chemotherapy-Induced Severe Neutropenia: A Pooled Analysis in Individual Patients Data with Advanced Lung Cancer. Lung Cancer.

[5] Danski M.T.R., Johann D.A., Vayego S.A., de Oliveira G.R.L., Lind J. (2016). Complicações Relacionadas Ao Uso Do Cateter Venoso Periférico: Ensaio Clínico Randomizado. Acta Paul. Enferm..

[6] Dimauro G., Guarini A., Caivano D., Girardi F., Pasciolla C., Iacobazzi A. (2019). Detecting Clinical Signs of Anaemia From Digital Images of the Palpebral Conjunctiva. IEEE Access.

[7] van de Weerdt E.K., Biemond B.J., Baake B., Vermin B., Binnekade J.M., van Lienden K.P., Vlaar A.P.J. (2017). Central Venous Catheter Placement in Coagulopathic Patients: Risk Factors and Incidence of Bleeding Complications. Transfusion.

[8] Rodriguez-Calero M.A., Fernandez-Fernandez I., Molero-Ballester L.J., Matamalas-Massanet C., Moreno-Mejias L., de Pedro-Gomez J.E., Blanco-Mavillard I., Morales-Asencio J.M. (2018). Risk Factors for Difficult Peripheral Venous Cannulation in Hospitalised Patients. Protocol for a Multicentre Case–Control Study in 48 Units of Eight Public Hospitals in Spain. BMJ Open.

[9] Kuderer N.M., Dale D.C., Crawford J., Cosler L.E., Lyman G.H. (2006). Mortality, Morbidity, and Cost Associated with Febrile Neutropenia in Adult Cancer Patients. Cancer.

[10] Baker M.J., Hussain S.R., Lovergne L., Untereiner V., Hughes C., Lukaszewski R.A., Thiéfin G., Sockalingum G.D. (2016). Developing and Understanding Biofluid Vibrational Spectroscopy: A Critical Review. Chem. Soc. Rev..

[11] Bunaciu A.A., Fleschin Ş., Hoang V.D., Aboul-Enein H.Y. (2017). Vibrational Spectroscopy in Body Fluids Analysis. Crit. Rev. Anal. Chem..

[12] Jöbsis F.F. (1977). Noninvasive, Infrared Monitoring of Cerebral and Myocardial Oxygen Sufficiency and Circulatory Parameters. Science.

[13] Chaves R.C.d.F., Tafner P.F.d.A., Chen F.K., Meneghini L.B., Corrêa T.D., Rabello R., Cendoroglo M., dos Santos O.F.P., Serpa A. (2019). Near-Infrared Spectroscopy Parameters in Patients Undergoing Continuous Venovenous Hemodiafiltration. Einstein.

[14] Vigo F., Tozzi A., Disler M., Gisi A., Kavvadias V., Kavvadias T. (2022). Vibrational Spectroscopy in Urine Samples as a Medical Tool: Review and Overview on the Current State-of-the-Art. Diagnostics.

[15] Hasan M.K., Aziz M.H., Zarif M.I.I., Hasan M., Hashem M., Guha S., Love R.R., Ahamed S. (2021). Noninvasive Hemoglobin Level Prediction in a Mobile Phone Environment: State of the Art Review and Recommendations. JMIR mHealth uHealth.

[16] Fisher R.A. (1936). The use of multiple measurements in taxonomic problems. Ann. Eugen..

[17] Cortes C., Vapnik V. (1995). Support-Vector Networks. Mach. Learn..

[18] Breiman L. (2001). Random Forests. Mach. Learn..

[19] Rosenblatt F. (1958). The Perceptron: A Probabilistic Model for Information Storage and Organization in the Brain. Psychol. Rev..

[20] Mannino R.G., Myers D.R., Tyburski E.A., Caruso C., Boudreaux J., Leong T., Clifford G.D., Lam W.A. (2018). Smartphone App for Non-Invasive Detection of Anemia Using Only Patient-Sourced Photos. Nat. Commun..

[21] Mantovani A., Cassatella M.A., Costantini C., Jaillon S. (2011). Neutrophils in the Activation and Regulation of Innate and Adaptive Immunity. Nat. Rev. Immunol..

[22] Xiong S., Dong L., Cheng L. (2021). Neutrophils in Cancer Carcinogenesis and Metastasis. J. Hematol. Oncol..

[23] Kim S.-M., Kim Y.-J., Kim Y.-J., Kim W.-Y. (2022). Prognostic Impact of Neutropenia in Cancer Patients with Septic Shock: A 2009–2017 Nationwide Cohort Study. Cancers.

[24] Gould Rothberg B.E., Quest T.E., Yeung S.J., Pelosof L.C., Gerber D.E., Seltzer J.A., Bischof J.J., Thomas C.R., Akhter N., Mamtani M. (2022). Oncologic emergencies and urgencies: A comprehensive review. CA Cancer J. Clin..

[25] Antoniadi A.M., Du Y., Guendouz Y., Wei L., Mazo C., Becker B.A., Mooney C. (2021). Current Challenges and Future Opportunities for XAI in Machine Learning-Based Clinical Decision Support Systems: A Systematic Review. Appl. Sci..

[26] Kariyawasam T.N., Ciocchetta S., Visendi P., Soares Magalhães R.J., Smith M.E., Giacomin P.R., Sikulu-Lord M.T. (2023). Near-infrared spectroscopy and machine learning algorithms for rapid and non-invasive detection of Trichuris. PLoS Negl. Trop. Dis..

[27] Sharma V.J., Adegoke J.A., Fasulakis M., Green A., Goh S.K., Peng X., Liu Y., Jackett L., Vago A., Poon E.K.W. (2023). Point-of-care detection of fibrosis in liver transplant surgery using near-infrared spectroscopy and machine learning. Health Sci. Rep..

[28] Martin F.L. (2023). Translating Biospectroscopy Techniques to Clinical Settings: A New Paradigm in Point-of-Care Screening and/or Diagnostics. J. Pers. Med..

