# Supplementary material for: Near-Infrared Spectroscopy with Supervised Machine Learning as a Screening Tool for Neutropenia
Source: J Pers Med. 2023 Dec 21;14(1):9. doi: 10.3390/jpm14010009 (PMC10817549; doi:10.3390/jpm14010009)
Supplement: Supplementary file 1 [file jpm-14-00009-s001.zip › jpm-2714244-supplementary.pdf]

Supporting Information

1. Outlier Detection

Hotelling's T^2 x Q residuals are statistical techniques used in multivariate analysis to detect outliers and assess the quality of a statistical model. Hotelling's T^2 statistic is a multivariate generalization of Student's t-test. It measures how far the sample mean vector of a multivariate dataset is from the population mean vector, accounting for the correlation structure between variables. Q residuals are used to identify univariate outliers in a multivariate dataset. It measures the Mahalanobis distance of individual observations from the centroid (mean vector) of the dataset, considering the correlation between variables.

1.1 Hotteling T^2 x Q plot

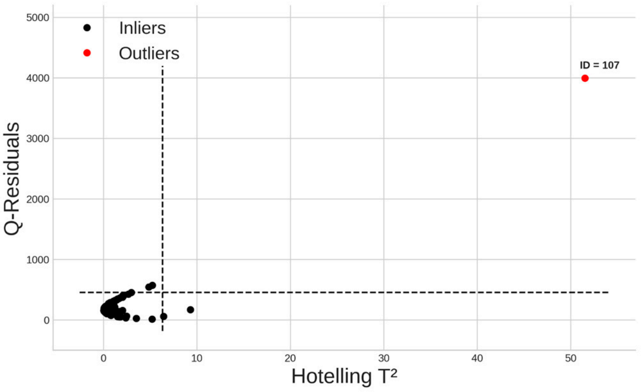

Figure S1. Hotelling T2 × Q plot evidencing one outlier (patient id = 107).

1.2 Outlier spectra

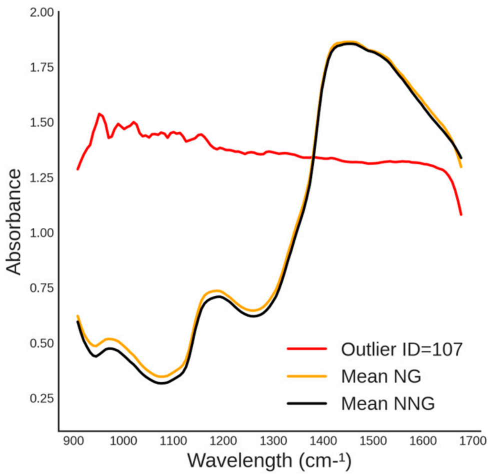

Figure S2. Outlier spectra versus average spectra per group (NG × NNG).
